# Supplementary material for: The ALS/FTD-related C9orf72 hexanucleotide repeat expansion forms RNA condensates through multimolecular G-quadruplexes
Source: Nat Commun. 2023 Dec 13;14:8272. doi: 10.1038/s41467-023-43872-1 (PMC10719400; doi:10.1038/s41467-023-43872-1)
Supplement: Supplementary file 1 — Supplementary Information [file 41467_2023_43872_MOESM1_ESM.pdf]

Supplementary Information

Figure S1: (GGGGCC)$_{n}$ forms multiple molecularity species. a. (GGGGCC)$_{n}$ FAM labelled AGE – FAM labelled (GGGGCC)$_{n}$ was annealed at 1 µM under G4-forming conditions (KCl 500 mM). Three distinct bands can be detected, respectively corresponding to bi-molecular and tetra-molecular species, with higher molecular weight species formed at higher repeat lengths, while lower molecularity species are more prominent at lower repeat lengths. **b. (GGGGCC)$_{n}$ FAM labelled AGE** – FAM labelled (GGGGCC)$_{n}$ was annealed at 350 µM under G4-forming conditions (KCl 500 mM). No distinct bands can be observed due to sample aggregation but is evident how higher repeat lengths lead to higher molecularity species.

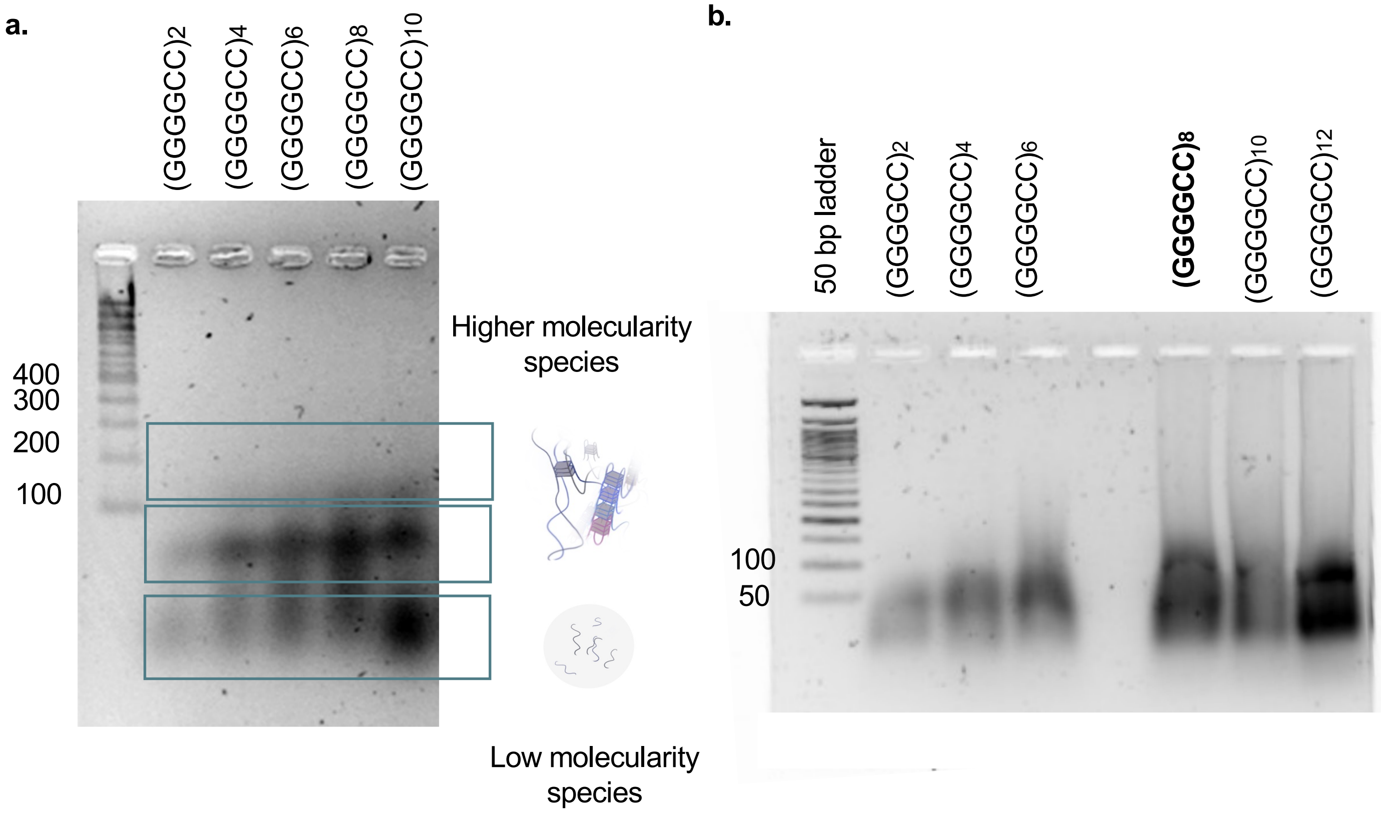

Supplementary Information

Figure S2: Carboxyfluorescein labelling of (GGGGCC)$_n$ aggregates. a. (GGGGCC)$_6$ FAM staining experiment – (GGGGCC)6 was annealed (50% FAM labelled) in mG4s forming conditions. Brightfield and fluorescence channel overlayed. **b. (GGGGCC)6 FAM staining experiment fluorescence images**– (GGGGCC)6 was annealed (50% FAM labelled) in mG4-forming conditions. **c. (GGGGCC)$_{10}$ FRAP experiment.** – (GGGGCC)10 was annealed (10% FAM labelled) in mG4-forming conditions. Upon laser excitation FAM labelled strands photobleached, leading to a local reduction of fluorescence intensity. The photobleached strands did not diffuse within the reset of the aggregate after 30 minutes, indicating a solid-like state. 10 μm scalebar.

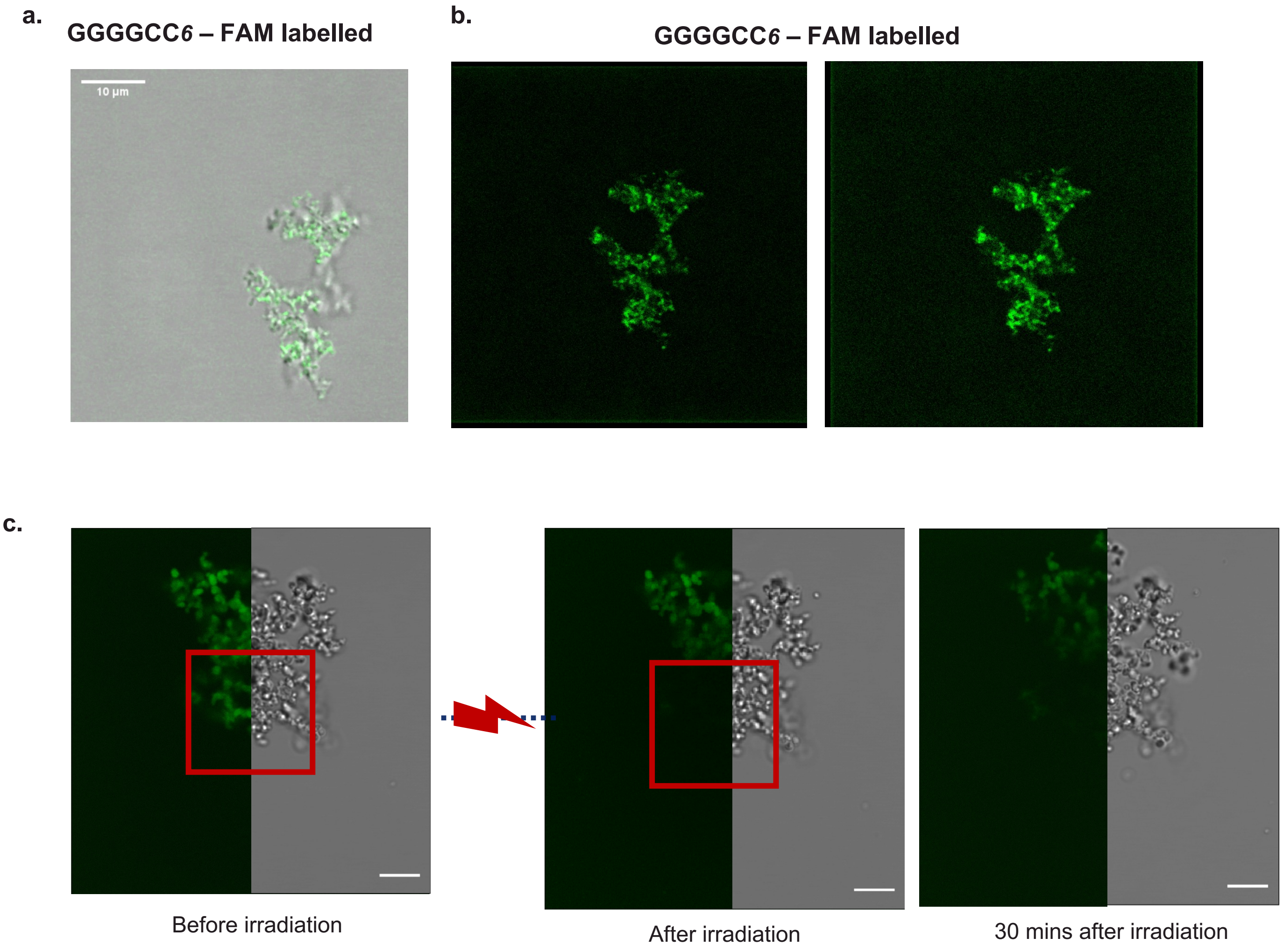

Figure S3: Phase Diagram with brightfield microscopy images.

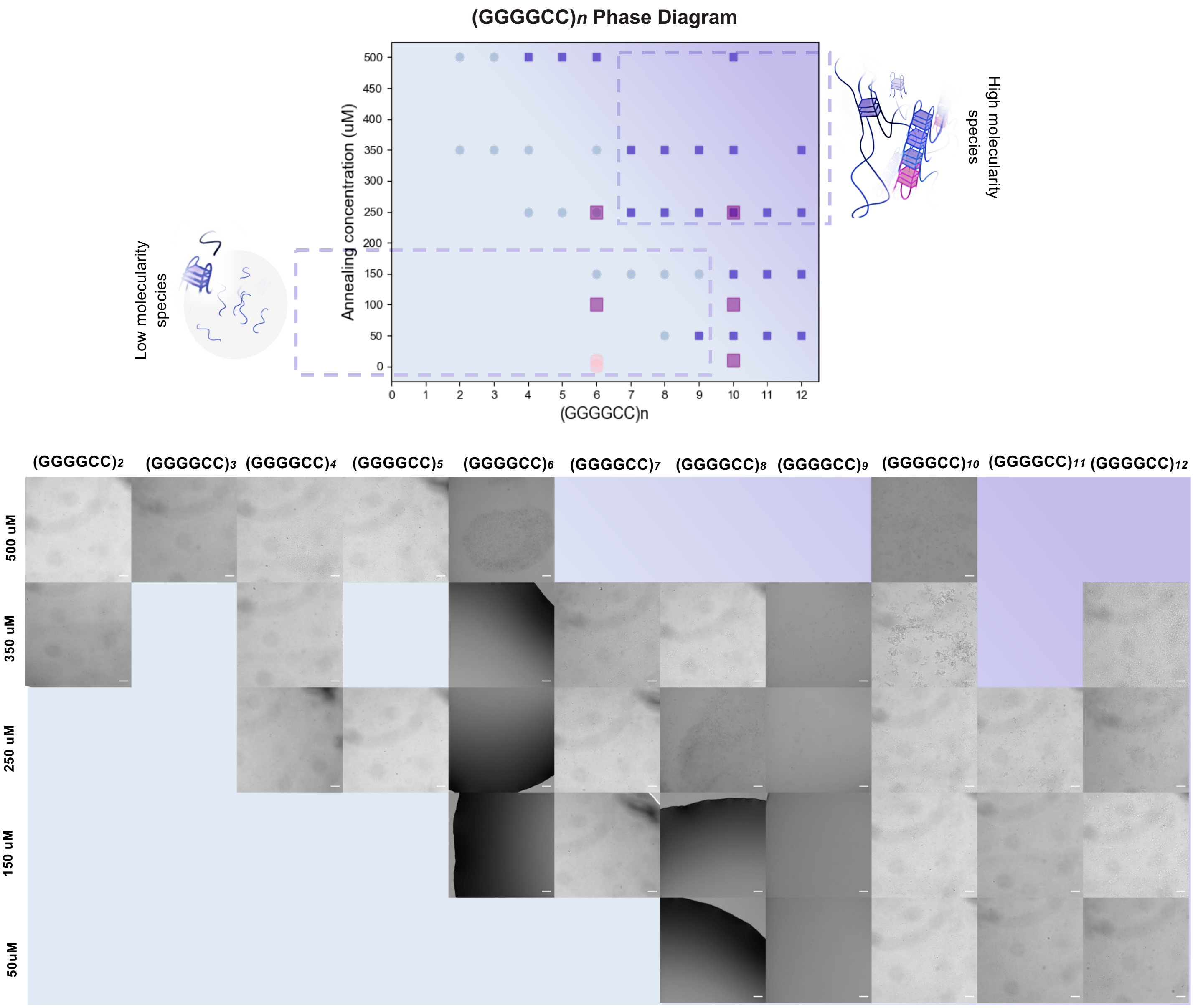

Figure S4: dsDNA condensates control for NMM staining and photooxidation experiments - a. Sequences used to build DNA condensates. **b.** Brightfield imaging and NMM fluorescent channel of the dsDNA condensates. No NMM fluorescence is observed in presence of dsDNA. **c.** dsDNA nanostar control for the photooxidation experiment. 10 µm scalebar.

a.

Name	Sequence (5'-3')
N4_ core1	**GATCGC**CGCCGCAATCACGCGCGTGCTCGGCGCCAGCA GTCCTGGCG
N4_core2	**GATCGC**CGCCAGGACTGCTGGCGCCGTCGCTTCTCTTCA TAACAACG
N4_core3	**GATCGC**CGTTGTTATGAAGAGAAGCGTCGCTCTGGCAC AGGTGTACG
N4_core4	**GATCGC**CGTACACCTGTGCCAGAGCGTGCACGCGCGTG ATTGCGGCG
L_AA1	**GCGATC**CGCAAACCAGCAAGCTCACG
L_AA2	**GCGATC**CGTGAGCTTGCTGGTTTGCG

b.

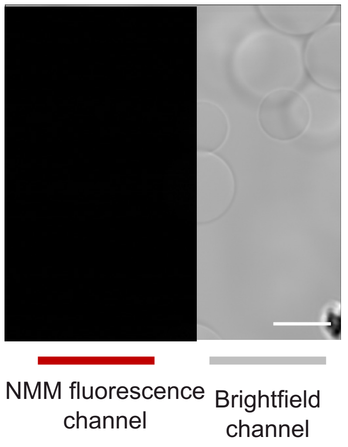

c.

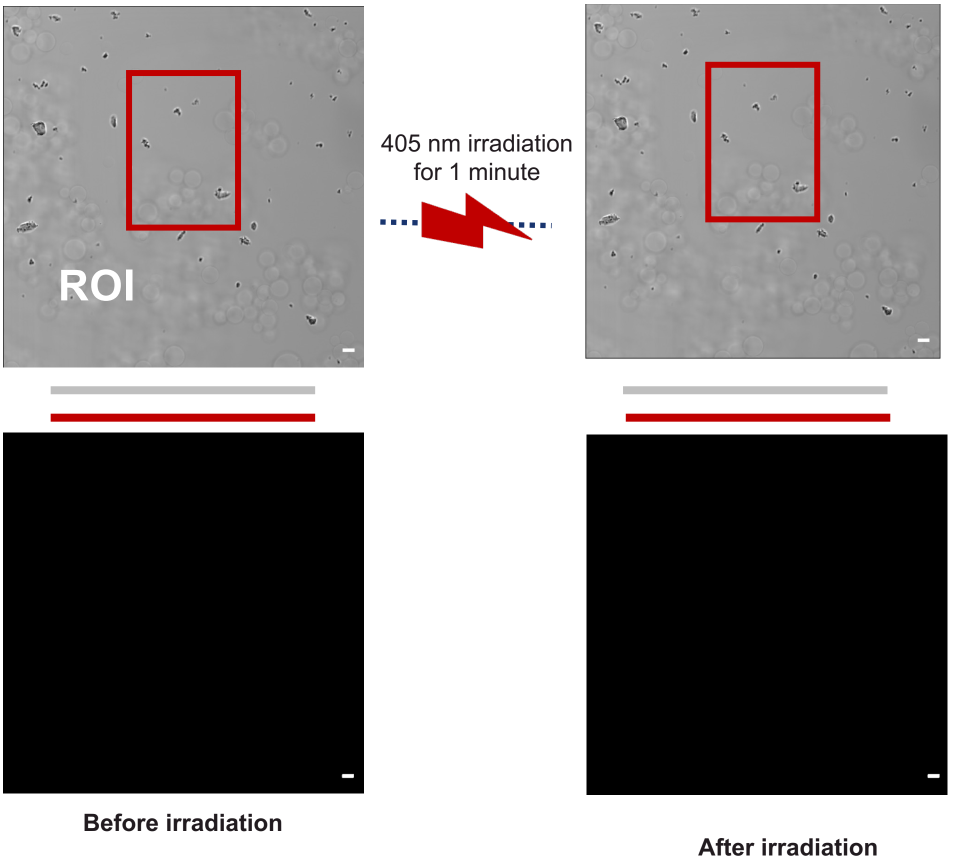

Figure S5: **DNA $(GGGGCC)_{11}$ aggregates form at physiological concentrations of KCl.** Brightfield imaging in of 500 µM $(GGGGCC)_{11}$ annealed with 100/300/500 mM KCl. 50 µm scalebar.

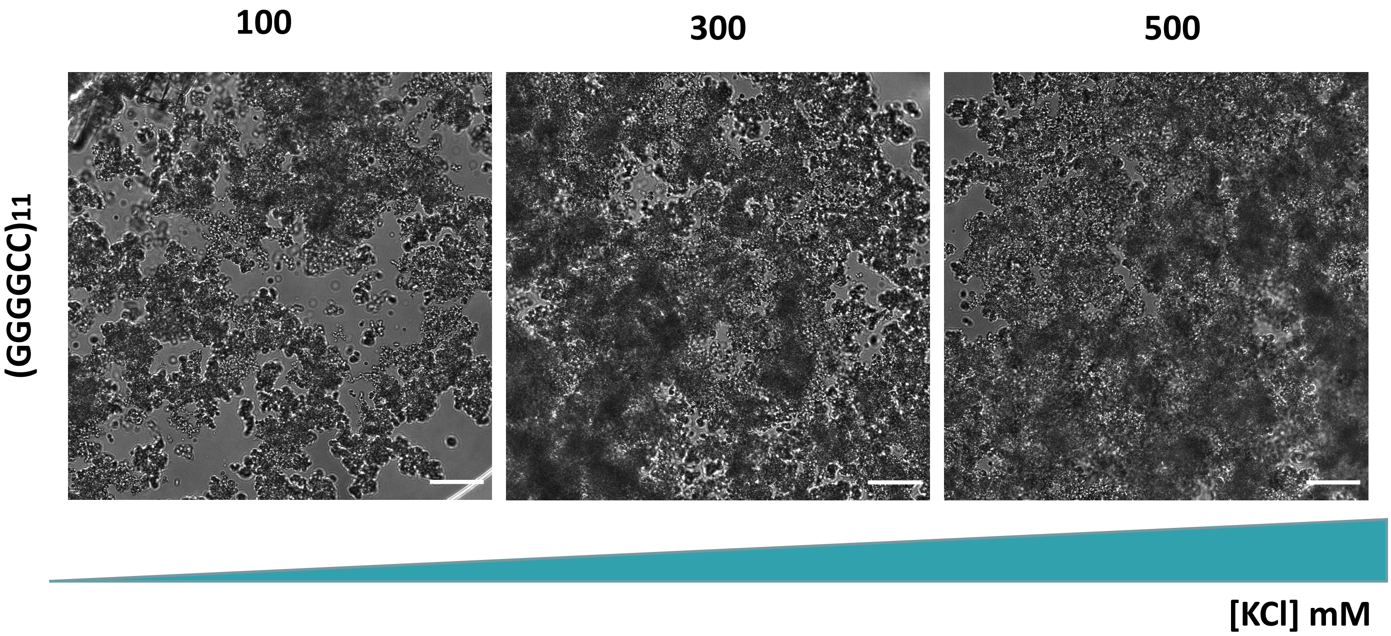

Figure S6: DNA $(GGGGCC)_{11}$ aggregates form in absence of the crowding agent PEG. Brightfield imaging of $(GGGGCC)_{11}$ annealed under mG4-forming conditions at 500 µM. On the right, a faster cooling rate is used in presence of the PEG crowding agent. On the left, a slower cooling rate in absence of PEG. (See methods for details) 50 µm scalebar.

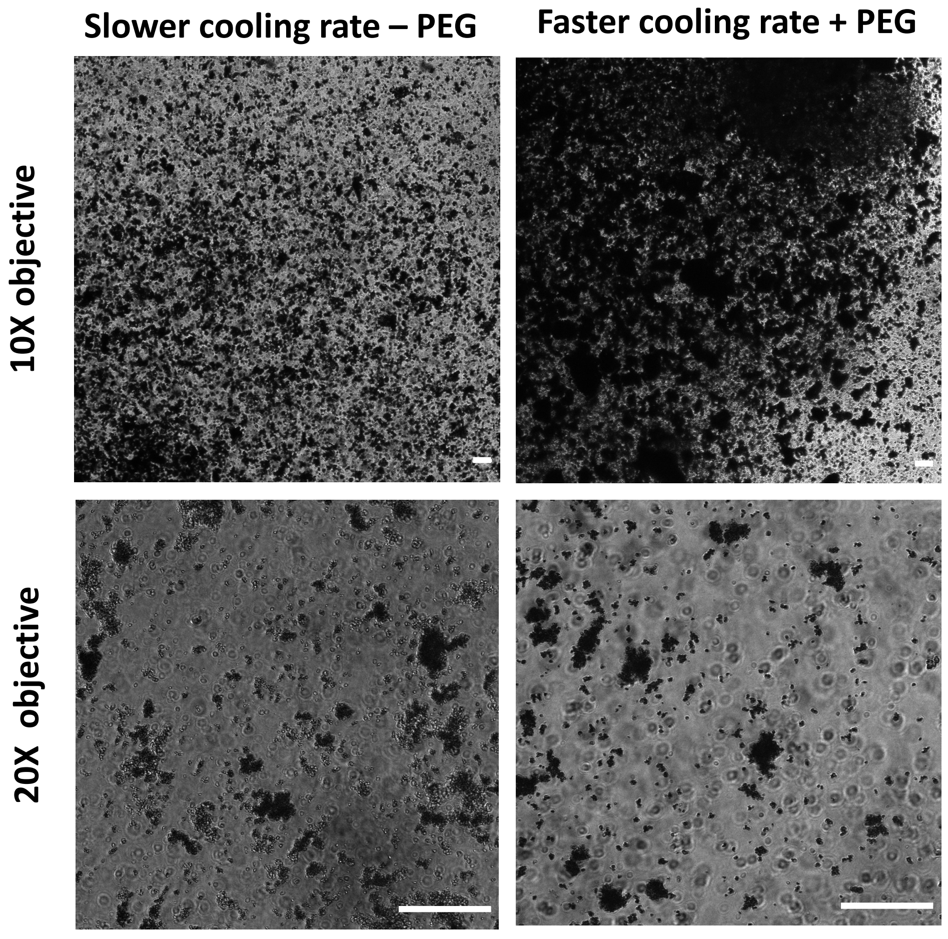

Figure S7: (GGGGCC)*n* forms lower molecularity species in presence of PDS. a. G4-ligand experiment agarose gel electrophoresis – SYBR safe stain. (GGGGCC)10 was annealed under mG4-forming conditions. Control samples in absence of PDS present higher molecularity species that appear as smearing in the gel. **b. G4-ligand experiment agarose gel electrophoresis – NMM stain**. (GGGGCC)10 was annealed under mG4-forming conditions. All samples containing G4-forming DNA appear stained by the probe.

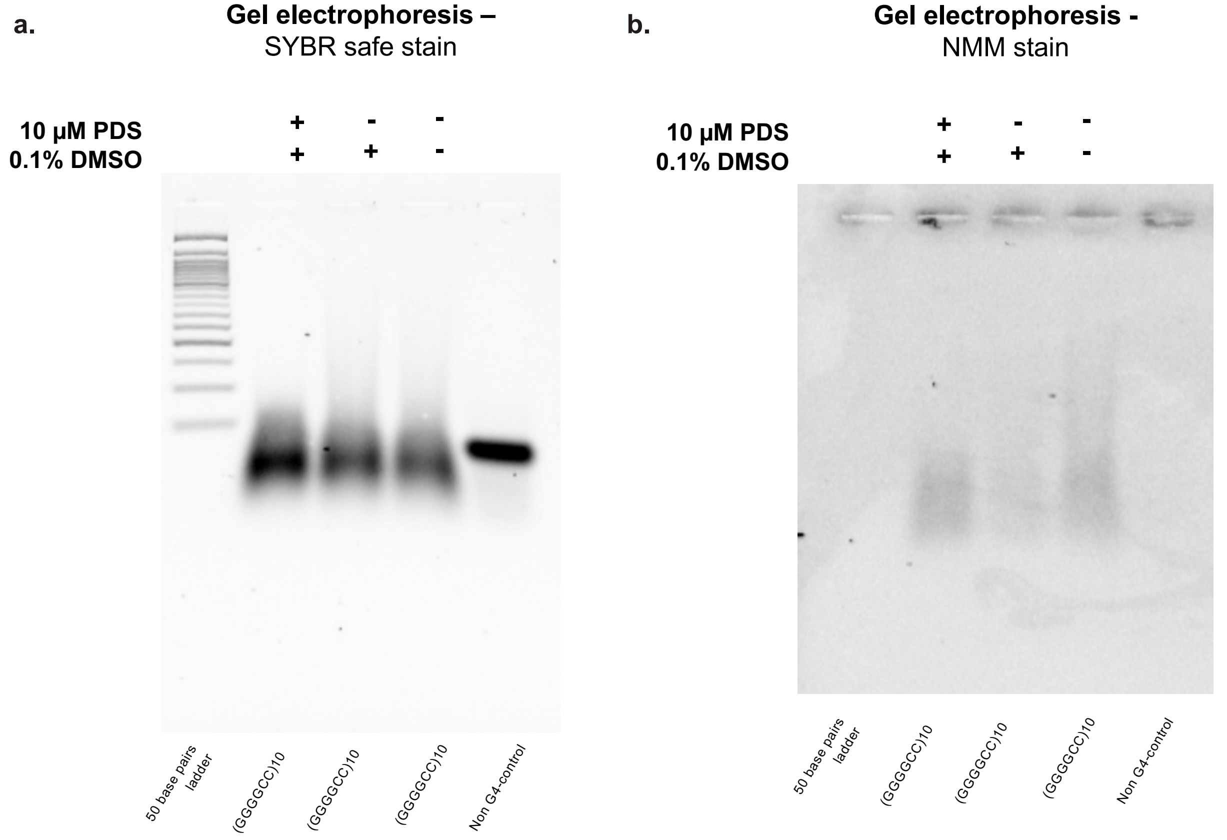

Figure S8: r(GGGGCC)*n* aggregates. Brightfield images of r(GGGGCC)n annealed at different concentrations.

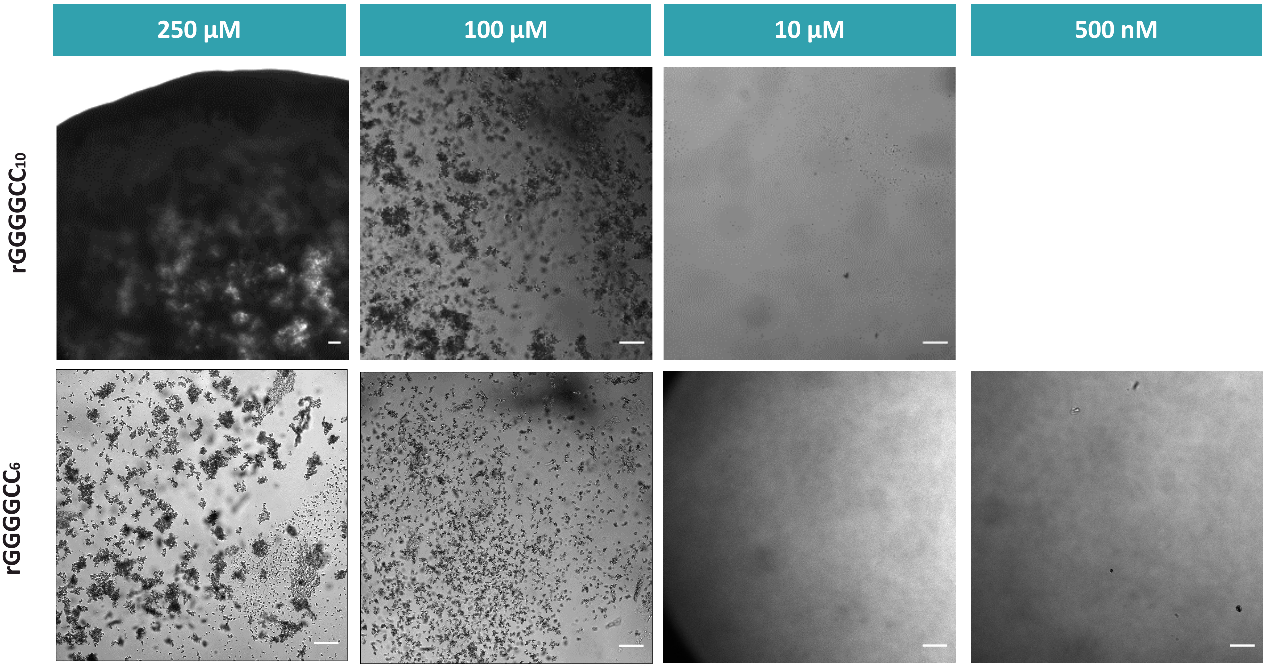

Figure S9: TDP-43 RRM1-2 after purification. a. SDS-PAGE gel and expected molecular weight form amino acid sequence. **b.** ESI-MS spectra after deconvolution.

a.

SDS-PAGE of TDP-43 RRM1-2 post size-exclusion chromatography (SEC)

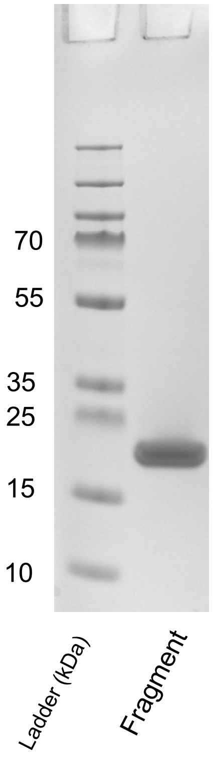

Amino acid sequence of RBDs:

KTSDLIVLGLPWKTTEQDLKEYFSTFGEVLMVQVKKDLKTGHSKG
FGFVRFTEYETQVKVMSQRHMIDGRWCDCKLPNSKQSQDEPLR
SRKVFVGRCTEDMTEDELREFFSQYGDVMDVFIPKPFRAFAFVTF
ADDQIAQSLCGEDLIIKGISVHISNAEPKHNSNRQ

Average M.W.: 19396.89219 ±1.31502

Monoisotopic M.W.: 19384.64622

b.

ESI-MS of TDP-43 RRM1-2 post size-exclusion chromatography (SEC)

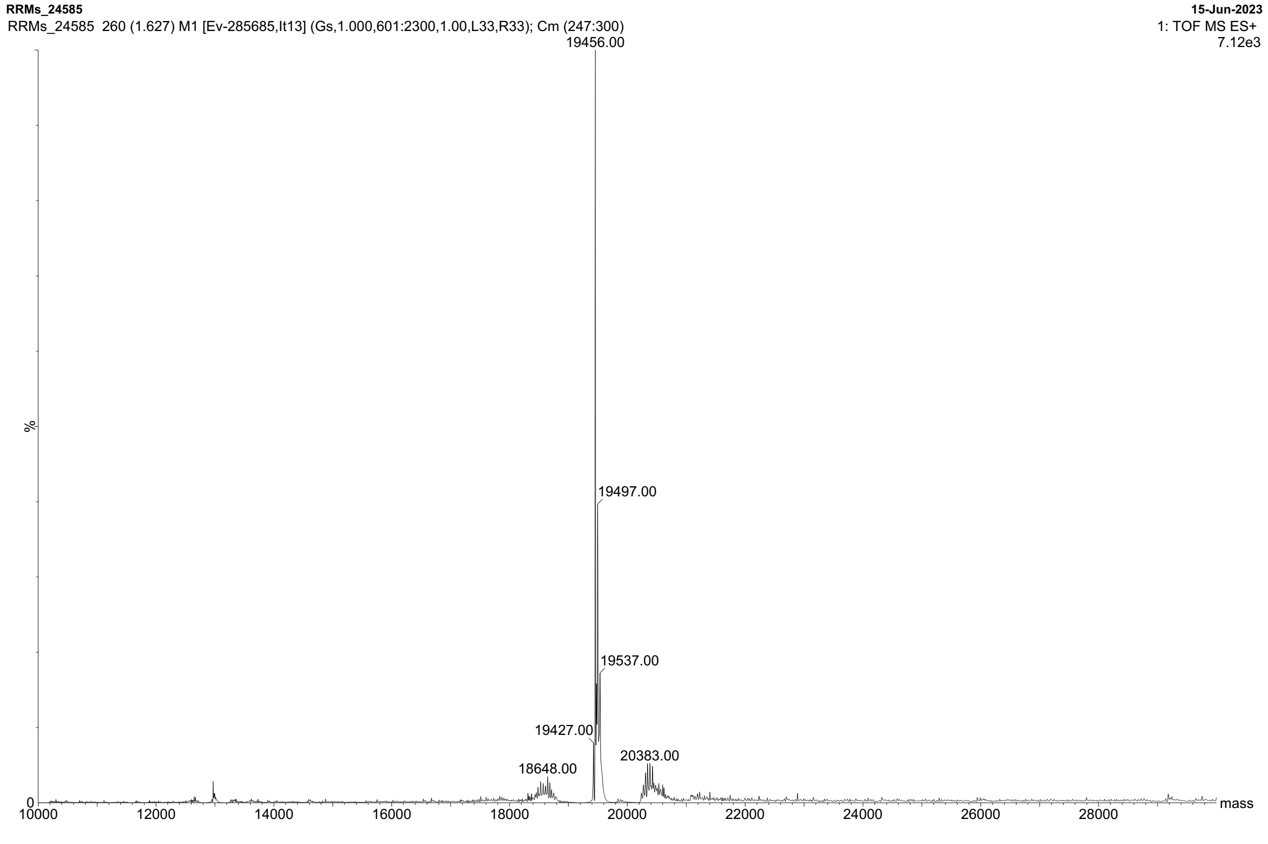

Figure S10: $(GGGGCC)_{11}$ in presence of TDP-43. a. $(GGGGCC)_{11}$ and TDP-43 aggregates. Confocal images of $(GGGGCC)_{11}$ annealed at 100 µM (FAM label) and incubated for three days with 10 µM TDP-43 RBD (Alexa 633 label). **b. $(GGGGCC)_{11}$ and TDP-43 aggregates NMM-stained.** 100 µm scalebar.

a.

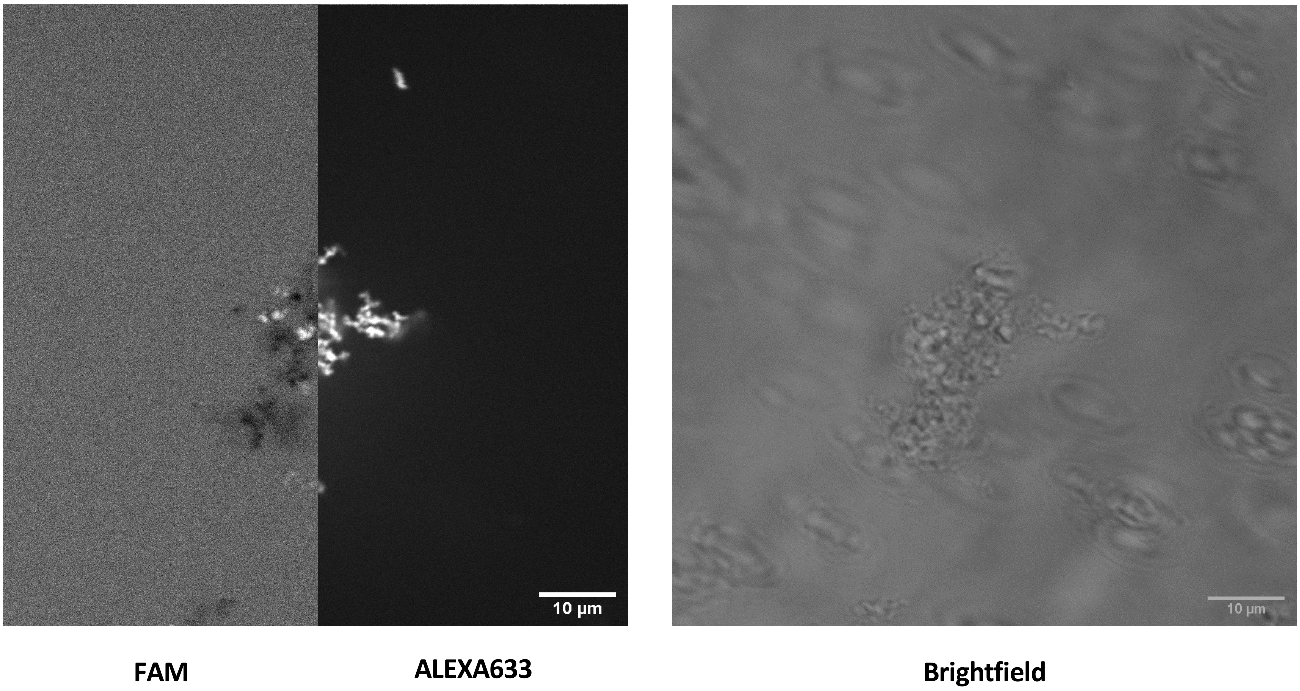

b.

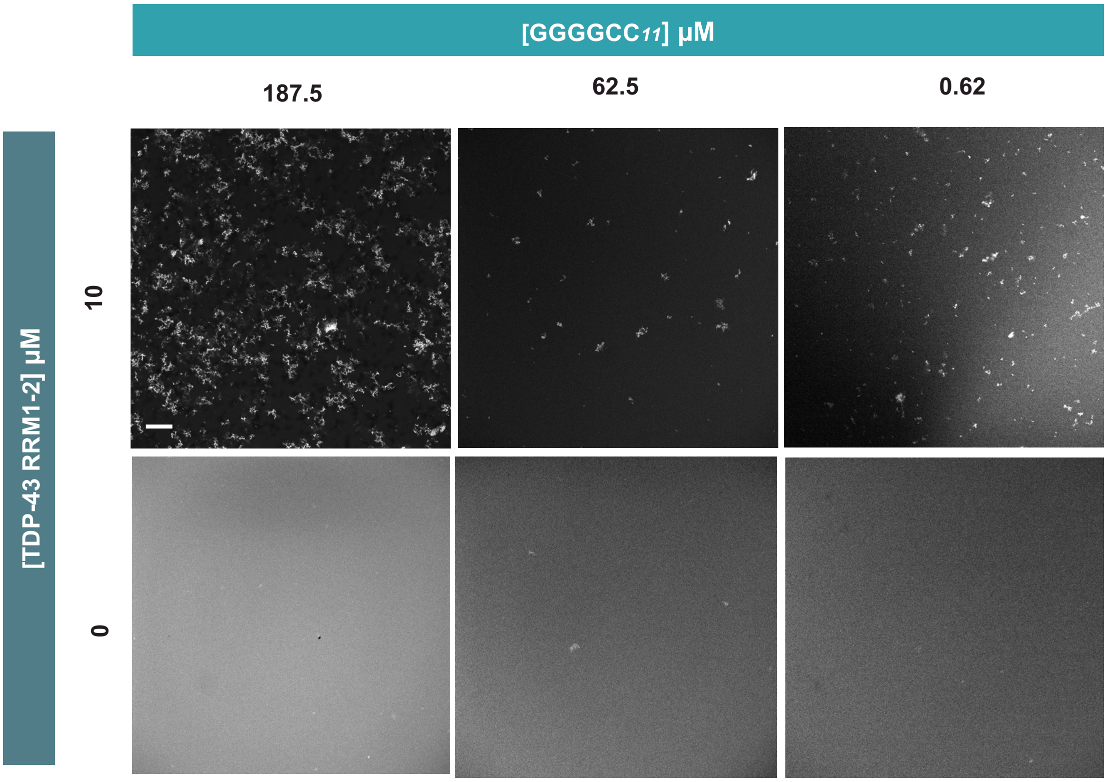

Figure S11: TEM images TDP-43 RRM1-2 aggregates. TEM images confirm that smaller aggregates can be seen beyond the resolution of a confocal microscope. These aggregates are still larger in presence of the DNA component.

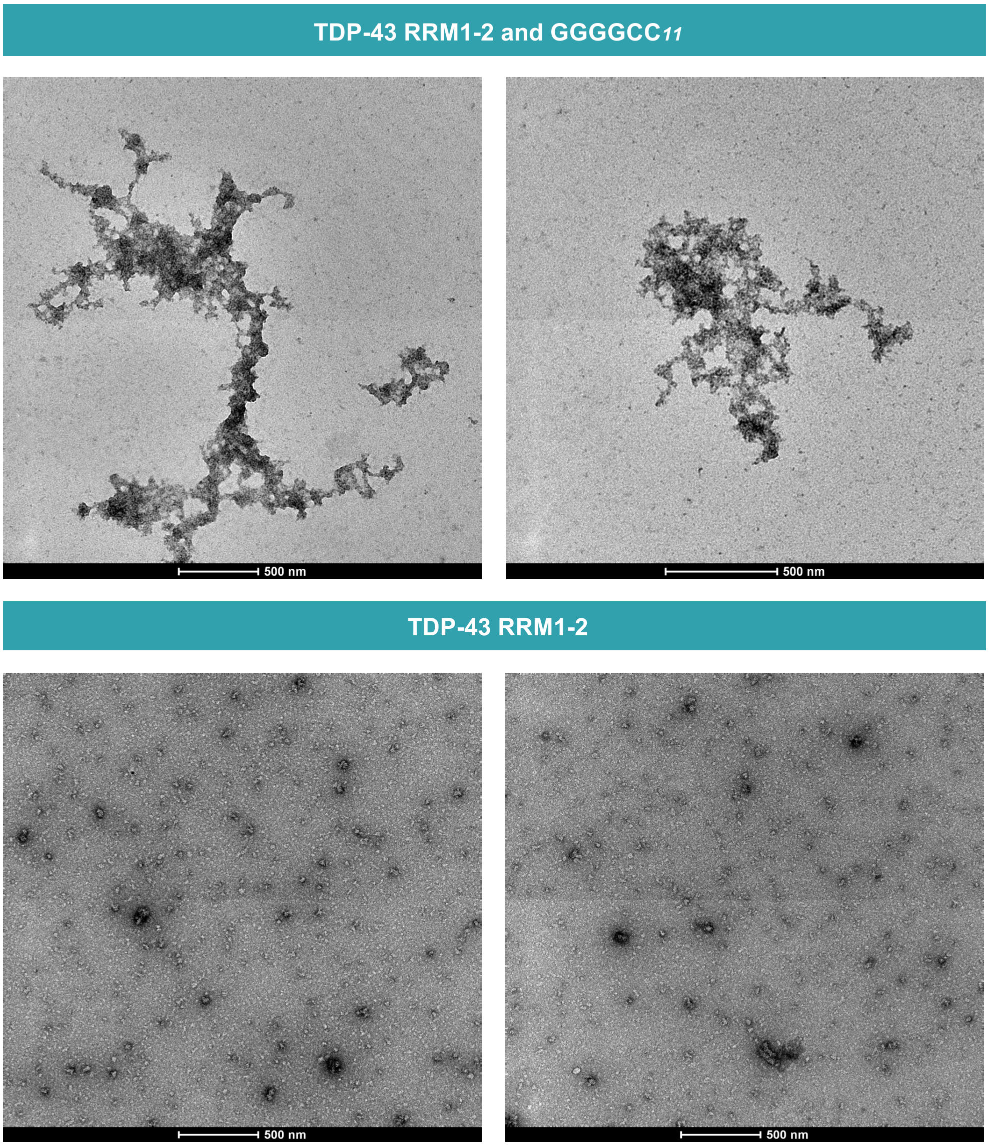

Figure S12: NMM is efficiently incorporated into iPSC derived motor neurons. a. Confocal representative images of iPSC derived motor neurons stained with neuronal marker β-Tubulin III (green) and nuclear dye DRAQ5 (red). Scale bar: 50 μm. **b.** Confocal representative images of motor neurons treated with DMSO and labelled with nuclear stain DRAQ5 (red). DMSO-treated control and C9orf72 mutant motor neurons exhibit no autofluorescence in the NMM spectrum. Scale bar: 50 μm. **c.** Quantification of the NMM signal intensity in DMSO- and NMM-treated control and C9orf72 cells. NMM is efficiently incorporated into iPSC derived motor neurons and specifically detected within its emission spectrum. P-value from two-way ANOVA with Tukey correction for multiple comparisons, p = 1.5673e-7 for CTRL+DMSO vs CTRL+NMM and p = 1.1517e-8 for C9+DMSO vs C9+NMM. **d.** Quantification of the percentage of neurons containing NMM foci in DMSO- and NMM-treated control and C9orf72 cells. Cells treated with NMM exhibit positive NMM foci across the whole population. P-value from two-way ANOVA with Tukey correction for multiple comparisons, p = 2.7e-14 for both CTRL+DMSO vs CTRL+NMM and C9+DMSO vs C9+NMM. **e**. Comparison of NMM foci intensity between NMM-treated control and C9orf72 cells. No difference in NMM intensity is observed between the two populations. P-value = 0.2753 from unpaired two-tailed t test. **f.** Comparison of NMM foci size between NMM-treated control and C9orf72 cells. No difference in NMM foci size is observed between the two populations. P-value = 0.9452 from two-tailed Mann-Whitney U test. Data for panels C-F are from n = 2 healthy and n = 2 mutant cell lines examined over 4 repeats with 9 fields of view per repeat and presented as mean ± SD, ****p < 0.0001.

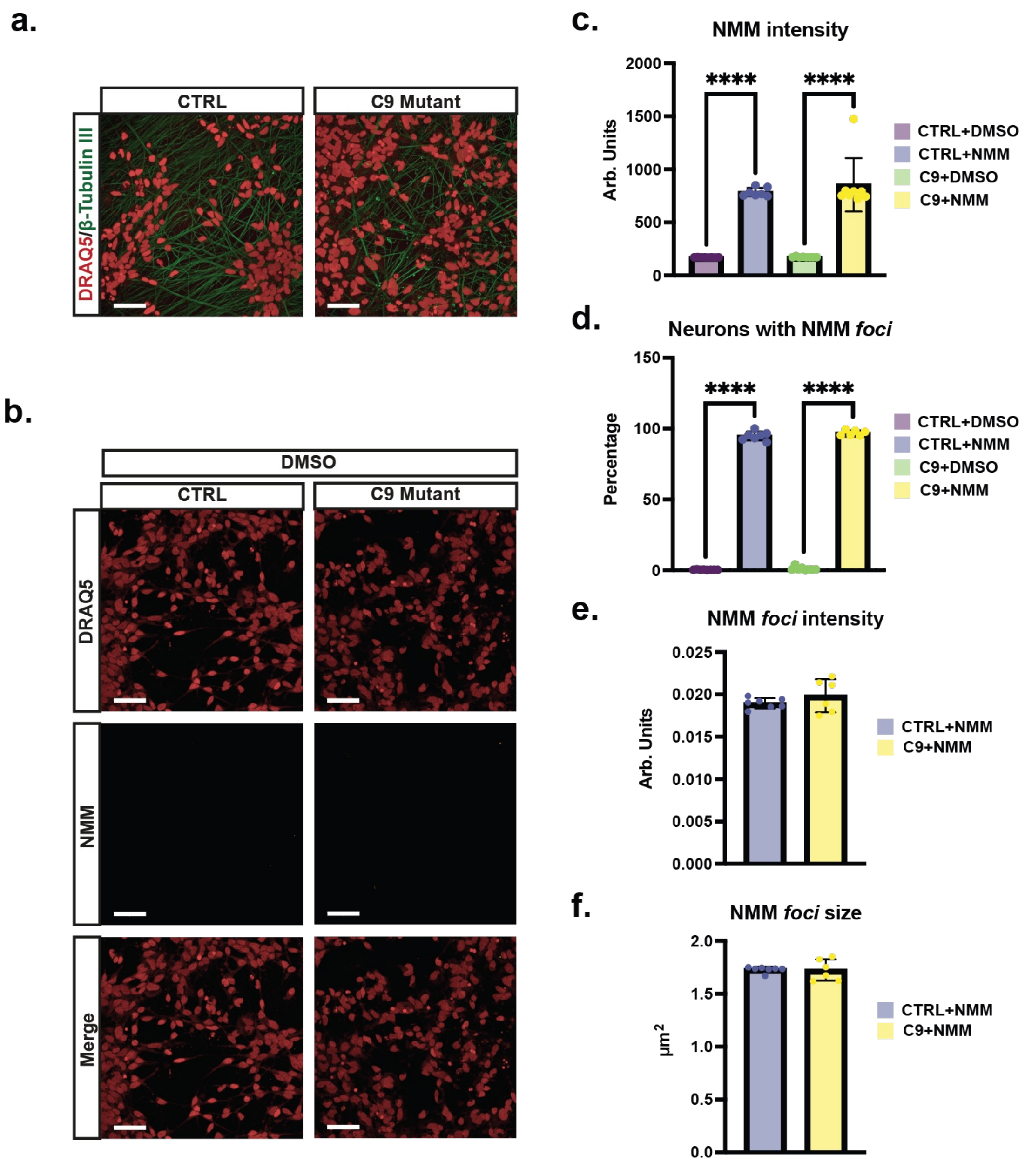

Figure S13: Flow cytometry data for iPSC-derived motor neurons treated with DMSO and NMM. a. Representative flow cytometry density plots for DMSO and NMM treatments in control and C9orf72 mutant cells. The plots indicate efficient incorporation of NMM into treated cells and minimal autofluorescence; **b.** DMSO and NMM treatment fluorescence histograms, where control (Left) and mutant (Middle) samples are compared individually, demonstrating that NMM was incorporated into the cells and the fluorescence is recorded in the characteristic NMM spectrum; Merged graph (Right) of DMSO and NMM fluorescence histograms in control and mutant cells. The intensity recorded in the mutant cells does not differ to that of their control counterparts**.**

a.

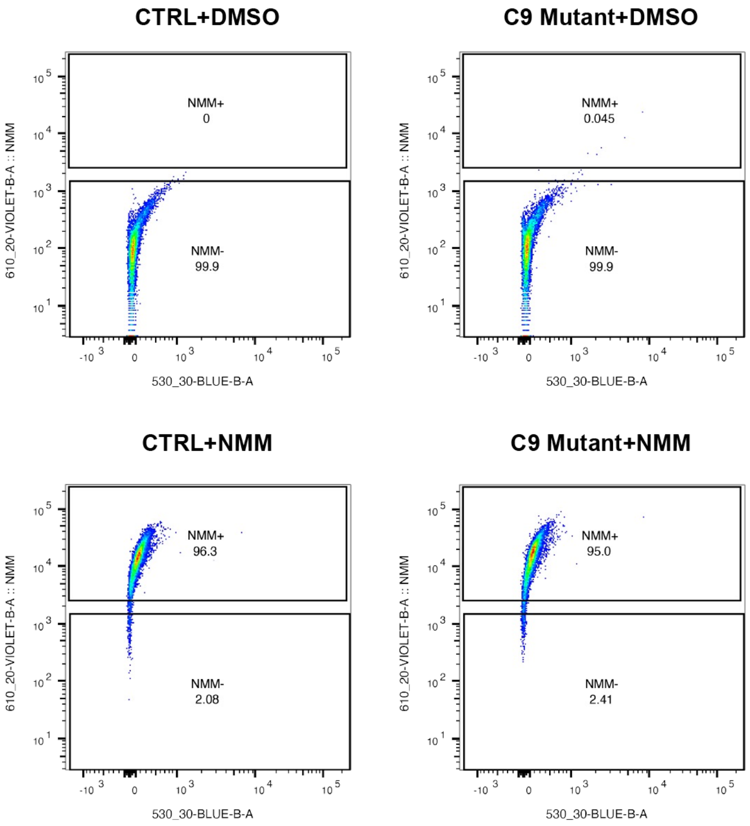

b.

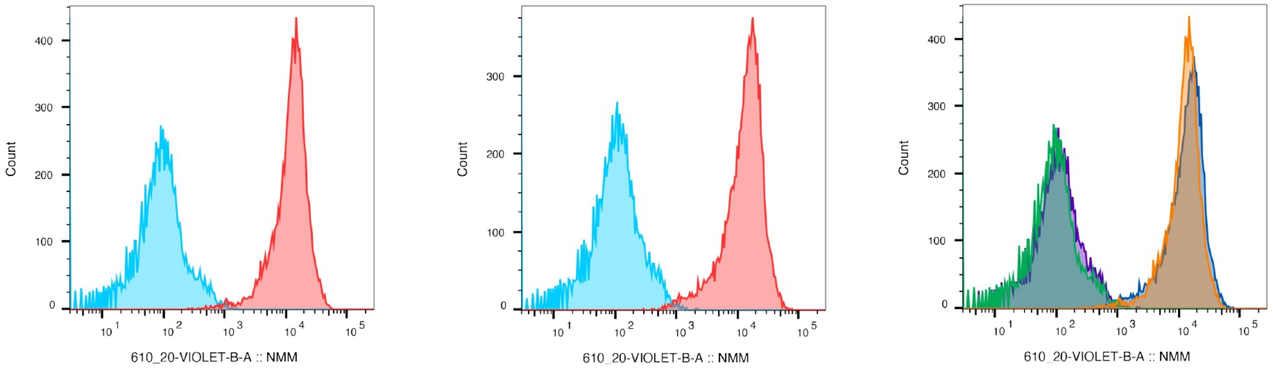

Sample Name	Count	Mean : 610_20-VIOLET-B-A
CTRL + 10μM NMM	10812	13693
CTRL+ DMSO	10972	125

Sample Name	Count	Mean : 610_20-VIOLET-B-A
C9orf72 Mutant + 10μM NMM	10987	15164
C9orf72 Mutant + DMSO	11081	145

Sample Name
CTRL + 10μM NMM
CTRL+ DMSO
C9orf72 Mutant + 10μM NMM
C9orf72 Mutant + DMSO
