# Supplementary material for: The ALS/FTD-related C9orf72 hexanucleotide repeat expansion forms RNA condensates through multimolecular G-quadruplexes
Source: Nat Commun. 2023 Dec 13;14:8272. doi: 10.1038/s41467-023-43872-1 (PMC10719400; doi:10.1038/s41467-023-43872-1)
Supplement: Supplementary file 3 — Reporting Summary [file 41467_2023_43872_MOESM3_ESM.pdf]

nature portfolio

Corresponding author(s): Dr Marco Di Antonio, Dr Lorenzo Di Michele and Prof Rickie Patani

Last updated by author(s): Nov 8, 2023

Reporting Summary

Nature Portfolio wishes to improve the reproducibility of the work that we publish. This form provides structure for consistency and transparency in reporting. For further information on Nature Portfolio policies, see our Editorial Policies and the Editorial Policy Checklist.

Statistics

For all statistical analyses, confirm that the following items are present in the figure legend, table legend, main text, or Methods section.

n/a	Confirmed	
☐	☒	The exact sample size (n) for each experimental group/condition, given as a discrete number and unit of measurement
☐	☒	A statement on whether measurements were taken from distinct samples or whether the same sample was measured repeatedly
☐	☒	The statistical test(s) used AND whether they are one- or two-sided<br>*Only common tests should be described solely by name; describe more complex techniques in the Methods section.*
☒	☐	A description of all covariates tested
☒	☐	A description of any assumptions or corrections, such as tests of normality and adjustment for multiple comparisons
☐	☒	A full description of the statistical parameters including central tendency (e.g. means) or other basic estimates (e.g. regression coefficient) AND variation (e.g. standard deviation) or associated estimates of uncertainty (e.g. confidence intervals)
☐	☒	For null hypothesis testing, the test statistic (e.g. F, t, r) with confidence intervals, effect sizes, degrees of freedom and P value noted<br>*Give P values as exact values whenever suitable.*
☒	☐	For Bayesian analysis, information on the choice of priors and Markov chain Monte Carlo settings
☒	☐	For hierarchical and complex designs, identification of the appropriate level for tests and full reporting of outcomes
☒	☐	Estimates of effect sizes (e.g. Cohen's d, Pearson's r), indicating how they were calculated

Our web collection on statistics for biologists contains articles on many of the points above.

Software and code

Policy information about availability of computer code

Data collection	LSRFortessa analyser operated by FACSDiva Software, LAS X Software (Leica Application Suite), ImageQuant LAS 4000, Tecnai Image Acquisition software
Data analysis	GraphPad Prism 9, Python (Jupiter Notebook 5.0.0), FlowJo, ImageJ2,

For manuscripts utilizing custom algorithms or software that are central to the research but not yet described in published literature, software must be made available to editors and reviewers. We strongly encourage code deposition in a community repository (e.g. GitHub). See the Nature Portfolio guidelines for submitting code & software for further information.

Data

Policy information about availability of data

All manuscripts must include a data availability statement. This statement should provide the following information, where applicable:

- Accession codes, unique identifiers, or web links for publicly available datasets
- A description of any restrictions on data availability
- For clinical datasets or third party data, please ensure that the statement adheres to our policy

The data generated in this study have been deposited in the Supplementary Information/Source Data file (https://doi.org/10.17863/CAM.104057) University of Cambridge Research Data Facility.

Research involving human participants, their data, or biological material

Policy information about studies with human participants or human data. See also policy information about sex, gender (identity/presentation), and sexual orientation and race, ethnicity and racism.

Reporting on sex and gender	Not applicable.
Reporting on race, ethnicity, or other socially relevant groupings	Not applicable.
Population characteristics	Not applicable.
Recruitment	Not applicable.
Ethics oversight	Not applicable.

Note that full information on the approval of the study protocol must also be provided in the manuscript.

Field-specific reporting

Please select the one below that is the best fit for your research. If you are not sure, read the appropriate sections before making your selection.

☒ Life sciences ☐ Behavioural & social sciences ☐ Ecological, evolutionary & environmental sciences

For a reference copy of the document with all sections, see nature.com/documents/nr-reporting-summary-flat.pdf

Life sciences study design

All studies must disclose on these points even when the disclosure is negative.

Sample size	No calculation was performed to determine the sample size of the biophysical characterization of (GGGGCC)n (n=2). As each annealing experiment requires large amount of expensive materials, after optimization of the protocol, each experiment in the manuscript was repeated a minimum of two times to ensure repeatability.<br>No calculation was performed to determine the sample size of the protein experiments in presence of (GGGGCC)n (n=2). As each annealing experiment requires large amount of expensive materials, after optimization of the protocol, each experiment in the manuscript was repeated a minimum of two times to ensure repeatability.<br>Cell studies: n = 2 healthy and n = 2 C9orf72 mutant cell lines were examined over 4 repeats to ensure that staining with NMM was repeatable. Samples were imaged and analyzed using high-throughput screening system (PerkinElmer). 9 fields of view were captured, providing an average of 400 cells per repeat, which is sufficient for statistical analysis
Data exclusions	No data was excluded from the analysis.
Replication	Biophysical characterization of (GGGGCC)n: Each experiment in the manuscript was repeated a minimum of two times to ensure repeatability. In particular, the authors would like to point out that due to the experimental conditions of the DNA annealing experiment, a significant amount of sample-to-sample variation can be present due to evaporation of the samples. This was accounted by utilising high volumes of buffer for the samples preventing evaporation (as noted in the methods section).<br>Protein studies: Each sample was run in biological duplicates to ensure repeatability.<br>Cell studies: n = 2 healthy and n = 2 C9orf72 mutant cell lines were examined over 4 repeats to ensure that staining with NMM was repeatable. Samples were imaged and analyzed using high-throughput screening system (PerkinElmer). 9 fields of view were captured per repeat.
Randomization	To reduce experimental bias in the biophysical characterization of the (GGGGCC)n aggregates and the protein studies, samples were randomly numbered prior to micro graphs acquisition. Therefore, samples were imaged without prior
Blinding	To reduce experimental bias in the biophysical characterization of the (GGGGCC)n aggregates and the protein studies, samples were randomly numbered prior to micro graphs acquisition. The person acquiring the images did not know what they were observing. The only exception is that of the cellular studies, for which a larger sample size and additional controls were added to reduce human bias.

Reporting for specific materials, systems and methods

We require information from authors about some types of materials, experimental systems and methods used in many studies. Here, indicate whether each material, system or method listed is relevant to your study. If you are not sure if a list item applies to your research, read the appropriate section before selecting a response.

Materials & experimental systems

n/a	Involved in the study	
☐	☒	Antibodies
☐	☒	Eukaryotic cell lines
☒	☐	Palaeontology and archaeology
☒	☐	Animals and other organisms
☒	☐	Clinical data
☒	☐	Dual use research of concern
☒	☐	Plants

Methods

n/a	Involved in the study	
☒	☐	ChIP-seq
☐	☒	Flow cytometry
☒	☐	MRI-based neuroimaging

Antibodies

Antibodies used	Rabbit polyclonal anti-tubulin beta-III (#802001), BioLegend; used at 1:2000 in 0.15% Triton-X in PBS.
Validation	Each lot of this antibody is quality control tested by formalin-fixed paraffin-embedded immunohistochemical staining. This antibody is well characterized and highly reactive to neuron specific Class III ß-Tubulin (ßIII), but does not identify ß-tubulin found in glial cells. Antibody RRID: AB_2564645 (BioLegend Cat. No. 802001). Relevant citations: Avraham O, et al. 2020. Nat Commun. 3.854861111. ; Mickle AD, et al. 2019. Nature. 565:361.

Eukaryotic cell lines

Policy information about cell lines and Sex and Gender in Research

Cell line source(s)	C9N1: CS29iALS-C9nxx (catalogue) | CS29iALS-C9 (individual) | 47 (Age of donor) | Male (sex of donor)<br>C9N6: CS52iALS-C9nxx (catalogue) | CS52iALS-C9 (individual) | 49 (Age of donor) | Male (sex of donor)<br>CTRL4: In house (DOI: 10.1016/j.celrep.2017.05.024) |Control (individual) | 51(Age of donor) | Female (sex of donor)<br>CTRL5: CS0002iCTR-nxx (catalogue) I Control (individual) I 51 (Age of donor) I Male (sex of donor)
Authentication	Cell lines were generated in house or by Cedar-Sinai where the authentication was previously performed, including mophology, STR profiling and Karyotyping.
Mycoplasma contamination	Cell are negative for mycoplasma contamination. The lines were regularly tested for mycoplasma contamination
Commonly misidentified lines (See ICLAC register)	No commonly misidentified lines were used in the experiments.

Flow Cytometry

Plots

Confirm that:

☒ The axis labels state the marker and fluorochrome used (e.g. CD4-FITC).

☒ The axis scales are clearly visible. Include numbers along axes only for bottom left plot of group (a 'group' is an analysis of identical markers).

☒ All plots are contour plots with outliers or pseudocolor plots.

☒ A numerical value for number of cells or percentage (with statistics) is provided.

Methodology

Sample preparation	Motor neurons were dissociated into single cells using Accutase (Life Technologies). The cell suspensions were fixed using 4% paraformaldehyde for 15-20 minutes and then permeabilized using 0.3% Triton-X in PBS for 1 hour at room temperature. Subsequently, they were treated with either 10 µM NMM (Cambridge Bioscience) or 0.5% DMSO diluted in 0.15% Triton-X overnight at 4°C.
Instrument	The samples (10,000 cells/sample) were examined using a BD Fortessa analyser operated by the FACSDiva software (BD).
Software	FACSDiva and FlowJo software
Cell population abundance	The populations in question were analyzed and not sorted therefore the purity is not relevant.
Gating strategy	Cells were first gated with FSC and SSC to select a homogeneous population. Singlet cells were next determined by SSC-H vs. SSC-A. Finally, NMM+ and NMM- populations were identified against a DMSO-treated control, where the NMM+ is less than 0.1% in the positive gate.

☒ Tick this box to confirm that a figure exemplifying the gating strategy is provided in the Supplementary Information.
